# *Notes from the Field:* Dengue Outbreak — U.S. Virgin Islands, January 2024–June 2025

**DOI:** 10.15585/mmwr.mm7540a2

**Published:** 2026-10-15

**Authors:** Sarah Gallalee, Hannah M. Cranford, Andra Prosper, Annellie Gumbs, Valerie V. Mac, Jendai Richards, Aubrey Drummond, Laura E. Adams, Gilberto A. Santiago, Parker K. Acevedo, Marlon Lawrence, Lisa LaPlace Ekpo, Brett R. Ellis, Esther M. Ellis

**Affiliations:** ^1^Epidemic Intelligence Service, CDC; ^2^U.S. Virgin Islands Department of Health; ^3^Office of the Director, National Center for Emerging and Zoonotic Infectious Diseases, CDC; ^4^Division of Vector-Borne Diseases, National Center for Emerging and Zoonotic Infectious Diseases, CDC, Puerto Rico.

**Summary**

**What is already known about this topic?**

Dengue virus caused substantial disease outbreaks in the Americas in 2024.

**What is added by this report?**

During 2024–2025, the U.S. Virgin Islands (USVI) experienced its first dengue virus outbreak in approximately a decade, including 263 confirmed cases and the territory’s first confirmed dengue virus–associated death since 2005. The outbreak response included implementing diagnostic testing for dengue in USVI for the first time, clinical trainings on case management and testing, deployment of a web-based electronic reporting platform, territorywide vector control campaigns, and community outreach and education.

**What are the implications for public health practice?**

Maintaining laboratory testing, surveillance capacity, vector control, and workforce training between outbreaks can strengthen preparedness and response during future dengue outbreaks in USVI.

In 2024, multiple large outbreaks caused by dengue virus (DENV) occurred in the Americas (*1*). During January 2024–June 2025, the U.S. Virgin Islands (USVI) experienced its first dengue outbreak since 2012–2013 (*2*,*3*). DENV is an arbovirus spread by *Aedes* species mosquitoes. Infection is often asymptomatic, but approximately 25% of persons with DENV infection experience febrile illness and 5% experience severe disease; <1% of patients with dengue die (*4*). The median incubation period is 5–7 days (range = 3–10 days) (*5*). This report describes findings from the USVI outbreak investigation.

## Investigation and Outcomes

### Data Sources and Analyses

Cases were identified by notifiable laboratory and clinical reporting to USVI’s Department of Health (VIDOH) and investigated using the 2015 Council of State and Territorial Epidemiologists dengue case definition. VIDOH and CDC conducted a dengue surveillance evaluation and rapid clinical needs assessment. This activity was reviewed by CDC, deemed not research, and conducted consistent with applicable federal law and CDC policy.*

### Patient Characteristics

In total, 263 confirmed cases were reported during January 2024–June 2025, including 118 (45%) on St. Croix, 105 (40%) on St. Thomas, and 40 (15%) on St. John (Table). Annual incidence reached 2.5 per 1,000 population in 2024. Most patients (256; 97%) reported no travel during the 2 weeks preceding symptom onset. Median patient age was 44 years (range = 2 months–89 years); 146 (56%) were female. Fifteen patients (6%) were hospitalized; the average length of stay was 4.3 days (range = 2–14 days). One patient, a woman aged 63 years with diabetes mellitus, died. Hers was the first confirmed DENV-associated death since 2005. Among 105 cases with a known DENV serotype, 101 (96%) were DENV-2 and four (4%) were DENV-3; three of the four DENV-3 cases were among persons who reported recent travel to Puerto Rico.

**TABLE:** Number and percentage of confirmed dengue cases on St. Croix, St. John, and St. Thomas, by demographic characteristics, hospitalization status, and dengue virus test results of patients — U.S. Virgin Islands, January 2024–June 2025

Characteristic	No. (column %)			
	St. Croix	St. John	St. Thomas	Total
**Total (row %)**	**118 (44.9)**	**40 (15.2)**	**105 (39.9)**	**263 (100.0)**
**Date of symptom onset**				
Jan–Mar 2024	1 (0.8)	1 (2.5)	0 (—)	**2 (0.8)**
Apr–Jun 2024	0 (—)	12 (30.0)	23 (21.9)	**35 (13.3)**
Jul–Sep 2024	6 (5.1)	26 (65.0)	77 (73.3)	**109 (41.4)**
Oct–Dec 2024	64 (54.2)	1 (2.5)	5 (4.8)	**70 (26.6)**
Jan–Mar 2025	42 (35.6)	0 (—)	0 (—)	**42 (16.0)**
Apr–Jun 2025	5 (4.2)	0 (—)	0 (—)	**5 (1.9)**
**Age group, yrs**				
Median age (range)	52 (7–89)	46 (11–82)	36 (<1–82)	**44 (<1–89)**
<1–17	18 (15.3)	4 (10.0)	20 (19.0)	**42 (16.0)**
18–34	15 (12.7)	6 (15.0)	30 (28.6)	**51 (19.4)**
35–49	19 (16.1)	10 (25.0)	23 (21.9)	**52 (19.8)**
50–64	38 (32.2)	10 (25.0)	19 (18.1)	**67 (25.5)**
≥65	28 (23.7)	10 (25.0)	13 (12.4)	**51 (19.4)**
**Sex**				
Female	60 (50.8)	17 (42.5)	69 (65.7)	**146 (55.5)**
Male	57 (48.3)	23 (57.5)	36 (34.3)	**116 (44.1)**
Unknown	1 (0.8)	0 (—)	0 (—)	**1 (0.4)**
**Hospitalized**				
Yes	9 (7.6)	3 (7.5)	3 (2.9)	**15 (5.7)**
No	80 (67.8)	32 (80.0)	83 (79.0)	**195 (74.1)**
Unknown	29 (24.6)	5 (12.5)	19 (18.1)	**53 (20.2)**
Positive test type*				
Serum PCR or NAAT	14 (11.9)	32 (80.0)	72 (68.6)	**118 (44.9)**
NS1	49 (41.5)	0 (—)	0 (—)	**49 (18.6)**
IgM	43 (36.4)	7 (17.5)	30 (28.6)	**80 (30.4)**
Test type unavailable	12 (10.2)	1 (2.5)	3 (2.9)	**16 (6.1)**
**Identified DENV serotype**				
DENV-2	2 (1.7)	31 (77.5)	68 (64.8)	**101 (38.4)**
DENV-3^†^	2 (1.7)	0 (—)	2 (1.9)	**4 (1.5)**
Unknown	114 (96.6)	9 (22.5)	35 (33.3)	**158 (60.1)**

**Abbreviations**: DENV = dengue virus; IgM = immunoglobulin M; NAAT = nucleic acid amplification test; NS1 = nonstructural protein 1; PCR = polymerase chain reaction.

* If one patient received multiple positive test results, the test type with the highest certainty is listed in the table; PCR had the highest certainty followed by NS1, then IgM.

^†^ One patient with DENV-3 infection on St. Croix reported no recent travel. The other three of the four patients with DENV-3 infection reported recent travel to Puerto Rico.

### Surveillance Evaluation and Clinical Needs Assessment

During August and September 2024, a surveillance evaluation and clinical needs assessment identified two important barriers to dengue surveillance and case management. Clinicians reported infrequent clinical encounters with patients with suspected dengue during the preceding decade and described a critical need for training on dengue clinical management, testing, and reporting. In addition, no local diagnostic testing for dengue was available in the territory: all samples were sent off the islands for testing, which led to turnaround times of 1–2 months for results. To address these barriers, VIDOH visited 30 clinics and conducted 20 clinical trainings on case management, testing, and reporting during fall 2024 and launched a dengue telephone hotline number in October 2024, to provide additional guidance to medical providers. Advancements included implementation of dengue testing at the Territorial Public Health Laboratory in October 2024, with a reduction in turnaround times to <1 week, and deployment of a web-based electronic platform for disease reporting during fall 2024.

### Vector Control and Community Outreach

To reduce DENV transmission, VIDOH, with CDC and the Puerto Rico Vector Control Unit (in collaboration with CDC’s Puerto Rico–based dengue branch), conducted vector control and community outreach campaigns. Outreach included door-to-door campaigns in 25 communities with reported dengue caseloads, removal of standing water (256 household yards inspected for *Aedes aegypti* breeding sites), outdoor larvicide application with *Bacillus thuringiensis* subsp. *Israelensis*, a group of bacteria that produces toxins lethal to some mosquitoes (167 households), distribution of spatial repellents called passive emanators^†^ (approximately 900 devices distributed), distribution of bed nets for cribs (seven child care, orphanage, and maternal child health care facilities), and community outreach and education regarding dengue prevention (420 households, 54 schools, 10 public events, and four press conferences).

## Preliminary Conclusions and Actions

VIDOH launched a comprehensive response to the 2024–2025 dengue outbreak, successfully implementing dengue testing locally, leveraging partnerships to train staff members and conduct vector control interventions, and providing clinician and public education. Maintaining capacity for dengue case management, surveillance, and vector control is imperative to enabling rapid and scalable responses and preventing future dengue outbreaks and severe disease in USVI.

## Footnotes

* 45 C.F.R. part 46, 21 C.F.R. part 56; 42 U.S.C. Sect. 241(d); 5 U.S.C. Sect. 552a; 44 U.S.C. Sect. 3501 et seq.

† Passive emanators are low-maintenance devices that slowly release an insecticide (transfluthrin, which is registered with the Environmental Protection Agency) into the air using natural airflow. Because they require no heat or electricity, these devices provide continuous indoor protection against mosquito bites during the life of the device, which might help reduce the risk for acquiring mosquitoborne diseases.
